# PEEK Intraoral Scan Bodies—A Scoping Review

**DOI:** 10.3390/dj14040222

**Published:** 2026-04-09

**Authors:** Ioulianos Rachiotis, Aspasia Pachiou, Daniel S. Thoma, Nadja Naenni, Christos Rahiotis

**Affiliations:** 1 Dental School, National and Kapodistrian University of Athens, Thivon 2, 11527 Athens, Greece; sdn2100077@uoa.gr; 2 Clinic of Reconstructive Dentistry, Center for Dental Medicine, University of Zurich, Plattenstrasse 11, 8032 Zurich, Switzerland; aspasia.pachiou@zzm.uzh.ch (A.P.); daniel.thoma@zzm.uzh.ch (D.S.T.); nadja.naenni@zzm.uzh.ch (N.N.); 3 Department of Operative Dentistry, Dental School, National and Kapodistrian University of Athens, Thivon 2, 11527 Athens, Greece

**Keywords:** PEEK, Polyetheretherketones, intraoral scanners, scan body, dental implants, digital dentistry

## Abstract

**Background**: Accurate digital impressions are crucial for the long-term success of implant-supported prostheses, with scan bodies playing a pivotal role in transferring the implant position into the virtual model. Recent work has focused on PEEK (polyether-etherketone) scan bodies because their optical behavior may facilitate intraoral scanning; however, the breadth and quality of supporting evidence remain unclear. **Methods**: This scoping review followed PRISMA-ScR reporting guidelines and was registered in the Open Science Framework (OSF; Registration DOI 10.17605/OSF.IO/CU3V8). Pub-Med/MEDLINE, Embase, and Scopus were searched through September 2025. Eligible designs included in vitro studies, randomized trials, observational studies, and technical reports evaluating PEEK scan bodies in implant dentistry. Screening and data extraction were performed in duplicate, and findings were synthesized descriptively. **Results**: The search identified 227 records, and after screening, 31 studies met the inclusion criteria. Most studies were in vitro, with limited clinical evidence, and only one prospective clinical study was identified. Outcomes commonly addressed trueness, precision, scan time, and handling. Comparators varied (e.g., titanium, resin; splinted vs. unsplinted), and the results on accuracy were heterogeneous, with deviations typically within clinically acceptable limits (<100 µm). **Conclusions**: PEEK scan bodies are applicable for digital implant impressions. Clinical data are sparse, though, and methods vary. Controlled clinical studies are necessary to confirm the accuracy, reliability, and indications of this approach compared to titanium ISBs.

## 1. Introduction

Accurate implant impressions are crucial for the long-term success of implant-supported prostheses, as even minor discrepancies can compromise the fit of restorations and lead to prosthetic or biological complications [1,2,3,4].

Traditional impression methods have been widely used; however, these methods are technique-sensitive and prone to inaccuracies resulting from material expansion, shrinkage, or deformation [5]. To overcome these shortcomings, digital impressions have emerged as a reliable alternative, supported by the integration of intraoral scanners (IOS) and computer-aided design/manufacturing (CAD/CAM) technologies [6,7]. Digital workflows offer several advantages, including improved efficiency, fewer treatment steps, greater patient comfort, and the elimination of material-related errors [8,9]. Overall, digital workflows enable a more predictable transfer of implant position into the virtual environment. Systematic reviews further indicate that digital impressions are at least as accurate as conventional impressions, and in many cases demonstrate superior performance [10,11].

A central element in this process is the intraoral scan body (ISB), which is attached to the implant or abutment and scanned together with surrounding tissues. Its geometry is then matched with a digital library file to accurately reproduce the implant position within a virtual model [12,13,14].

The accuracy of intraoral scanning for implants depends on multiple factors, including ISB’s material, geometry, connection type, dimensions, torque, scanner characteristics, and operator experience [15,16,17]. Among the available materials, titanium and PEEK are the most frequently used for ISBs. Titanium offers durability and reusability, but its reflective surface may interfere with optical scanning, compromising accuracy [18,19]. PEEK, by contrast, is a non-reflective thermoplastic polymer with excellent biocompatibility and favorable optical behavior, making it highly suitable for intraoral scanning [20,21,22,23,24].

Despite its promise, the current body of evidence on PEEK ISBs is limited mainly to in vitro and scarce clinical data. In contrast, several systematic reviews have synthesized evidence on ISBs in general, highlighting factors such as design, material, geometry, splinting, and application protocols as key determinants of digital impression accuracy, though without a dedicated focus on PEEK [25,26,27,28,29,30].

Given these considerations, this scoping review aims to comprehensively map and synthesize the available evidence on the use of PEEK scan bodies in implant dentistry. The objectives are to summarize the current knowledge, identify methodological trends and limitations, and outline future directions to improve the clinical application and standardization of PEEK ISBs.

## 2. Materials and Methods

### 2.1. Protocol and Reporting

A scoping review was chosen due to the heterogeneity of the available evidence, which mainly consists of in vitro studies with different methodologies and outcome measures. This variability did not allow for a meaningful meta-analysis, making a scoping approach more suitable for mapping the existing evidence and identifying research gaps.

This scoping review followed PRISMA-ScR reporting guidelines, which provided a structured framework for all stages of the review process [31]. The PRISMA 2020 checklist is provided in Supplementary Table S1. The protocol was prospectively registered in the Open Science Framework (OSF; Registration DOI: 10.17605/OSF.IO/CU3V8) on 9 September 2025. No restrictions on publication status, year, or country of origin were applied.

### 2.2. Research Question and Framework

The primary research question of this scoping review was “What is the evidence regarding the accuracy of digital implant impressions using PEEK intraoral scan bodies compared to other materials?” Secondary objectives included identifying factors influencing accuracy, such as scan body material, geometry, torque application, sterilization/reuse, scanner type, and environmental conditions.

The PCC framework was applied:

- **Population (P):** Patients receiving dental implants, or implant models used in in vitro studies.
- **Concept (C):** Use of PEEK (polyetheretherketone) ISBs for digital impression procedures and intraoral scanning.
- **Context (C):** Implant dentistry workflows, including in vitro studies, clinical studies (prospective or retrospective), and technical reports.

### 2.3. Eligibility Criteria

- **Inclusion criteria:**
  ○ Studies evaluating PEEK ISBs in dental implantology.
  ○ Study types include in vitro studies, randomized controlled trials (RCTs), observational studies (both prospective and retrospective), and technical reports.
  ○ Publications from peer-reviewed journals.
  ○ Language: English.
  ○ Publication period: 2010–present.
- **Exclusion criteria:**
  ○ Studies focusing exclusively on ISBs made of other materials (e.g., titanium, resin, hybrid) without including PEEK.
  ○ Case reports, reviews, editorials, expert opinions, and conference abstracts without full text.
  ○ Animal studies.

### 2.4. Search Strategy

A comprehensive literature search was conducted in PubMed/MEDLINE, Embase, and Scopus to identify studies evaluating PEEK scan bodies in implant dentistry. The search strategies combined controlled vocabulary (MeSH/Emtree) and free-text terms related to material (PEEK, polyetheretherketone, high-performance polymers), scan bodies (scan body, scanbodies, scanning abutments, scan posts, scan flags, implant impression posts), and the digital workflow context (dental implants, intraoral scanning, digital impressions, CAD/CAM). The final search was conducted on 5 September 2025. The complete database-specific strategies for all three databases are provided in Appendix A (Supplementary Materials). Additionally, the reference lists of all included studies and relevant reviews were manually screened to identify any additional relevant publications.

### 2.5. Selection Process

- **Stage 1:** Titles and abstracts were screened independently by two reviewers (IR, AP).
- **Stage 2:** Full texts of potentially relevant articles were assessed against eligibility criteria.
- **Disagreements:** Resolved by discussion or consultation with a third reviewer (CR).

**Figure 1:**
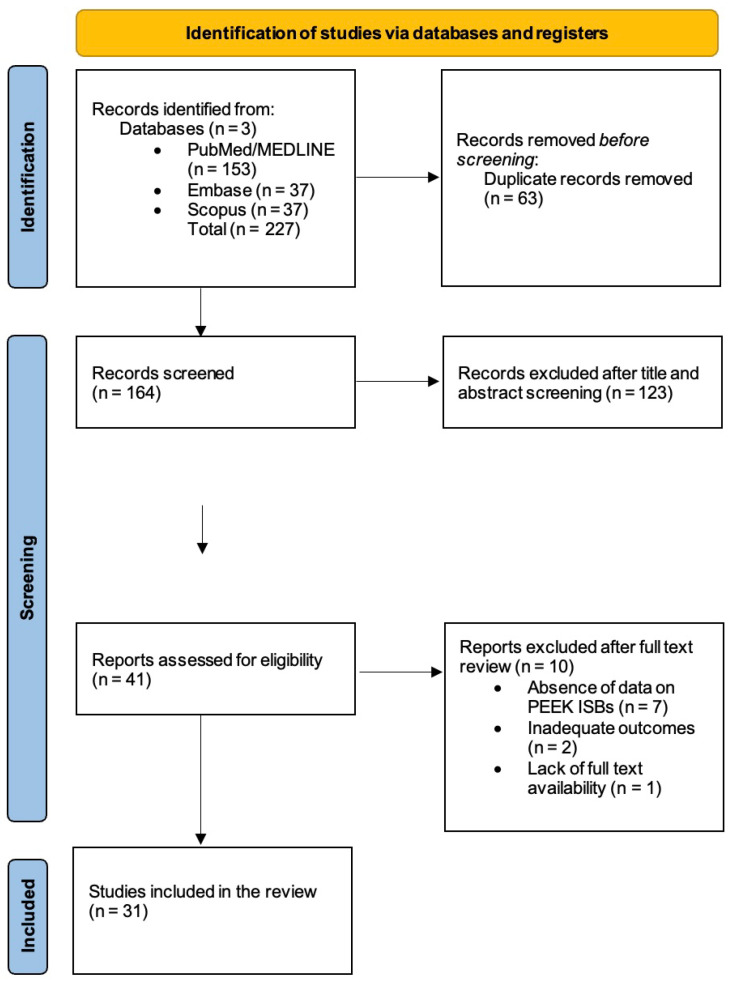
PRISMA 2020 flow diagram.

The process is presented in the PRISMA-ScR flow diagram (Figure 1).

### 2.6. Data Extraction

A standardized data extraction form was developed. Reference management and screening were performed using Rayyan (Qatar Computing Research Institute). The following items were collected:

- Author(s);
- Year;
- Study design (in vitro, RCT, observational study);
- Jaw/region;
- Type of edentulism;
- No. of implants;
- Implant system/connection;
- Scan body material;
- Control group;
- Type of intraoral/lab scanner used;
- Metrics;
- Measurement method for accuracy (e.g., trueness, precision, superimposition analysis);
- Key outcomes related to PEEK scan bodies.

### 2.7. Data Synthesis

Data were synthesized descriptively and presented in both tabular and narrative forms. Studies were grouped into in vitro and clinical categories. No formal risk-of-bias assessment or meta-analysis was conducted, consistent with the scoping review methodology. Quantitative synthesis was not feasible due to heterogeneity in study design, measurement parameters, and reporting metrics.

## 3. Results

### 3.1. Study Selection and Characteristics

The initial electronic database search identified 227 records, while no additional records were retrieved from grey literature or manual reference searching. After removing 63 duplicates, 164 records were screened by title and abstract. Of these, 41 full-text articles were assessed for eligibility, and 10 were excluded for reasons such as ineligible study design, absence of data on PEEK implant scan bodies, inadequate outcomes, or lack of full-text availability. A total of 31 studies met the inclusion criteria and were included in this scoping review. The study selection process followed the PRISMA-ScR framework and is summarized in the PRISMA flow diagram (Figure 1). Detailed characteristics and principal findings of all included studies are presented in Supplementary Table S2 [18,19,32,33,34,35,36,37,38,39,40,41,42,43,44,45,46,47,48,49,50,51,52,53,54,55,56,57,58,59,60], while the main comparative findings and factors affecting accuracy are summarized in Table 1 and Table 2.

The included studies were published between 2012 and 2025, comprising 30 in vitro studies and 1 prospective clinical study [49]. The majority of studies investigated fully edentulous arches (n = 18), whereas partial edentulism was evaluated in fewer studies [18,41,46,49,52,55,56,58]. Most investigations assessed PEEK implant scan bodies (ISBs) in direct-to-implant digital workflows, whereas a minority evaluated multi-unit abutment ISBs [47,50]. Comparative materials included titanium ISBs, hybrid PEEK–titanium ISBs, and 3D-printed resin prototypes. Commonly used intraoral scanners (IOS) were TRIOS 3/4 (3Shape A/S, Copenhagen, Denmark), Primescan (Dentsply Sirona, Bensheim, Germany), Omnicam (Dentsply Sirona, Bensheim, Germany), Medit i700 (Mesit Corp., Seoul, South Korea), CS3600 (Carestream Dental LLC, Atlanta, GA, USA), and iTero (Align Technology, Inc., San Jose, CA, USA). Reference datasets were typically obtained via coordinate measuring machines (CMMs) or desktop optical scanners.

Most studies focused on assessing trueness and precision, while secondary outcomes included scanning time, ease of handling, and the influence of scanner type or scan strategy.

### 3.2. Comparative Findings on PEEK and Titanium Scan Bodies

Studies directly comparing PEEK and titanium scan bodies showed heterogeneous findings [18,37,38,39,41,45,46,48,54,59,60]. Several in vitro studies reported higher trueness, precision, or dimensional stability for titanium scan bodies, particularly in complete-arch settings, after repeated sterilization or under higher tightening torque [37,38,39,45,48,54,59]. However, other studies have reported comparable or more favorable outcomes with PEEK under specific scanning conditions, including certain intraoral scanners and implant angulations [18,41,46,60]. Overall, the available evidence indicates that the effect of scan body material is not uniform but depends on interactions among the material, the scanner system, the scan body design, and the scanning conditions [18,37,45,46,59,60].

### 3.3. Factors Affecting Scan Accuracy

Across the included studies, scan accuracy was influenced by multiple factors beyond material alone. These included sterilization and reuse [38,39,41,42,54], tightening torque [39,48], scan body geometry and exposure [45,47,59], implant angulation [18,60], inter-implant distance and span length [49,50,53], scanner type [37,45,46,47,60], scan strategy [53], and environmental conditions such as saliva [59]. In general, longer spans and more complex scan configurations were associated with greater error accumulation, whereas controlled scanning conditions and optimized scan body design were associated with better accuracy [45,49,50,53,59]. The only included clinical study suggested that digital impressions with PEEK scan bodies were clinically acceptable for implant-supported prostheses up to three units in bounded edentulous saddles, although accuracy decreased with increasing span length [49].

To provide a consolidated overview, the main experimental and clinical findings regarding PEEK intraoral scan bodies are summarized in Table 2. This table highlights the principal factors influencing digital accuracy, such as material composition, reuse behavior, torque application, geometry, scanner type, and environmental conditions, and outlines their respective clinical and technical implications. Collectively, these findings emphasize that while PEEK ISBs generally achieve clinically acceptable accuracy, their performance depends strongly on standardized torque control, limited reuse cycles, optimized design geometry, and careful management of scanning conditions.

## 4. Discussion

This scoping review synthesised current evidence on PEEK ISBs used in implant digital workflows, focusing on material and geometric characteristics, reuse/sterilisation behaviour, and scanning accuracy.

### 4.1. Material and Reuse-Related Aspects

The material composition of scan bodies remains a critical factor influencing the accuracy of digital implant impressions [20]. Among the materials currently available, polyetheretherketone (PEEK) and titanium are the most widely used; however, comparative data across studies reveal inconsistent performance trends. Several investigations suggest that titanium scan bodies exhibit higher spatial accuracy under certain clinical conditions [61]. In contrast, others report no significant difference or even superior performance of PEEK in specific configurations. For instance, Soltan et al. observed that PEEK scan bodies achieved higher trueness at 30° and greater precision at 0°, despite similar overall 3D displacement (ΔR, *p* = 0.759) [60]. Likewise, Mahmoud Hashemi et al. found that PEEK outperformed titanium after 9 reuse cycles [41]. Collectively, these data suggest that no single material demonstrates consistent superiority across all conditions. Such findings indicate that accuracy cannot be attributed solely to material properties, but rather depends on a combination of optical reflectivity, mechanical fit, and surface characteristics that interact with scanner technology and environmental conditions.

The effects of sterilization and repeated reuse have also been extensively investigated, given their potential to alter material dimensions and surface integrity. PEEK scan bodies have shown greater susceptibility to deformation after multiple autoclave cycles than titanium, particularly at the abutment level (*p* < 0.01) [38]. Nonetheless, deviations for both materials remained below clinically relevant thresholds (<50 µm), and all surfaces were classified as “clinically adapted,” supporting the feasibility of controlled reuse. Similar observations were made by Aktas et al. and Bin Qasim et al., who found that sterilization-induced wear occurs regardless of the underlying material [34,62]. Significantly, mechanical factors such as tightening torque can further modulate dimensional stability. Morita et al. demonstrated that increasing torque from 10 to 35 Ncm significantly elevated displacement (*p* < 0.01), with titanium components showing greater deformation but more accurate replication of the abutment subsidence pattern, suggesting superior mechanical conformity under load [48].

Overall, these findings show that both material type and mechanical handling influence digital accuracy. Titanium exhibits higher mechanical resilience under load, whereas PEEK provides advantages in optical scanning and controlled reusability when maintained within defined sterilization cycles.

### 4.2. Geometry and Height/Exposure

Beyond material properties, the geometry and height of ISBs significantly influence the accuracy of digital implant impressions [27].

Several studies have further emphasized the impact of scan body geometry—including length, head design, and overall shape—on scanning performance. Simplified, shorter scan body designs were associated with higher trueness, whereas greater height or more complex geometries tended to compromise accuracy [63]. Consistent with these findings, Mohajerani et al. demonstrated that designs featuring rigid extensions or simplified head geometries improved both trueness and precision, underscoring the importance of optimizing ISB form to enhance digital accuracy [28].

Scan body geometry—particularly length and head design—plays a critical role in digital implant-level accuracy: simplified, shorter designs generally improve trueness, whereas greater height or geometric complexity can compromise it, and designs with rigid extensions or simplified geometries have shown superior trueness and precision [28,63]. That said, the influence of height is not universal; one study found supramucosal height did not significantly affect accuracy, suggesting height effects are design- and system-dependent [64]. Beyond geometry, implant placement level also contributes: tissue-level implants exhibited greater trueness and precision than bone-level implants (*p* < 0.05), indicating that placement level, alongside scan-body geometry/height, can influence digital impression accuracy [65].

The findings of the present review are broadly consistent with this interpretation: geometry and height materially affect accuracy, with simpler, well-indexed designs performing more reliably [47,58]. Cylindrical PEEK ISBs with flat reference surfaces enhanced rotational stability, and custom grooves improved scanning reliability. However, contradictory results have been found regarding the influence of height [51,59]. Taken together, the evidence suggests that optimizing scan-body design—particularly by achieving geometric simplicity and ensuring sufficient height for optical detection—remains crucial for maximizing scan accuracy.

### 4.3. Accuracy of PEEK Scan Bodies in Intraoral Scanning Workflows

The collected evidence indicates that PEEK scan bodies can achieve high accuracy when used under favorable conditions (e.g., single implants or limited spans with good exposure and straight angulation). Nevertheless, translating to complex clinical scenarios, such as multi-unit cases, angulated implants, edentulous arches, and long spans, remains less specific. A recent systematic review of intraoral scanning accuracy reported that while scan-body material is indeed essential, the evidence base remains limited, and outcomes vary with implant angulation and scanner type [27]. Thus, although PEEK shows promise, it cannot yet be deemed universally superior across all contexts, particularly in the absence of robust clinical trials.

To address challenges in extensive restorations, auxiliary scanning aids that mechanically link ISBs have emerged. A novel calibrated intraoral scan protocol (CISP) demonstrated greater accuracy than both standard intraoral scanning and scan aids alone, suggesting that structured, calibrated linking can mitigate cumulative error over longer spans [66]. Complementing this, full-arch edentulous investigations with PEEK scan bodies have shown significant vertical and horizontal deviations in tissue-level configurations; both tissue-level and bone-level tapered groups exhibited horizontal shifts and tilting, highlighting the sensitivity of full-arch accuracy to connection geometry and seating forces [67].

Environmental and geometric factors also modulate performance. Different ambient lighting conditions and color temperatures significantly affect intraoral scanning accuracy—an effect especially pertinent when attempting full-arch capture with PEEK scan bodies [68]. Similarly, the presence of saliva has been shown to adversely affect moisture control during scanning, which can affect the accuracy of digital implant transfer [59]. Additionally, a greater inter-implant distance increases the errors in trueness and precision. While these distortions were reported as not clinically significant within the studied ranges, they underscore the need for cautious span planning and error-control strategies [69].

In summary, PEEK scan bodies perform well under controlled, favorable conditions; however, accuracy in complex, full-arch scenarios depends on multiple interacting variables, including angulation, scanner choice, connection design and torque, ambient lighting, and inter-implant spacing. Protocols that stabilize and calibrate the scan (e.g., CISP with linked ISBs) appear promising for reducing cumulative error; however, there is a lack of high-quality clinical trials to confirm generalizability [27,66,67,68,69].

### 4.4. Clinical Implications and Recommendations

From a clinical point of view, the use of PEEK ISBs in digital implant workflows has several potential advantages: lower reflection on scanning surfaces, PEEK is radiolucent and less shiny than metal, better mechanical resilience to scratches or wear (in some models), and possibly cost benefits when reusable. However, given the heterogeneity of evidence and unknown long-term performance, clinicians should apply several practical strategies:

1. Select ISBs with sufficient exposure height so that the body is well above soft tissue level and accessible to the scanner tip. This enhances signal capture and reduces distortion.
2. Ensure firm, repeatable seating and tightening of the ISB using the manufacturer’s torque specification, to reduce micro-motion or mis-seating.
3. Avoid excessive reuse or sterilisation cycles of PEEK scan bodies beyond documented wear thresholds. Consider inspection or replacement after a defined number of cycles.
4. Control the scanning environment: dryness, limited saliva pooling, good access, minimal angulation and spacing, adequate operator training. These factors continue to influence accuracy significantly.
5. Limit span and complexity when possible: For long-span or complete-arch implant cases, or angulated implants, the risk of cumulative stitching error and ISB-related distortion increases; in these scenarios, the choice of scan body material is only one of many risk factors, and the workflow should be optimised.

From a practical perspective, PEEK scan bodies may be preferred in situations where optical scanability is critical, such as intraoral full-arch scanning with reflective environments. Conversely, titanium scan bodies may be advantageous when mechanical stability and resistance to deformation are prioritized, particularly under high-torque conditions or with repeated reuse.

Clinicians should therefore select scan body material based on the clinical scenario rather than assuming inherent superiority of one material over another.

### 4.5. Research Gaps and Future Directions

This scoping review reveals several gaps in the literature that warrant attention in future work:

- There is a lack of well-designed clinical studies that compare PEEK vs. titanium (or other materials) scan bodies in actual patient workflows, since the majority of current studies are in vitro.
- Longitudinal data linking scan body material to prosthetic outcomes (fit, misfit, mechanical complications, biological peri-implant responses) are essentially lacking.
- Studies investigating clinical reuse protocols of PEEK scan bodies under real-world conditions are limited.
- Standardisation of reporting metrics for trueness/precision (e.g., consistent units, reference models, exposure heights, reuse cycles) would enable future meta-analysis and more robust evidence synthesis.

Addressing these gaps will be essential to establish evidence-based guidelines for material selection and clinical application of PEEK ISBs.

### 4.6. Strengths of This Review

This scoping review was prospectively registered, PRISMA-ScR-compliant, and based on a comprehensive multi-database search with duplicate screening and standardized data extraction to ensure methodological rigor. Its focused scope on PEEK scan bodies, rather than on generic ISBs, allows for material-specific insights. At the same time, the synthesis integrates key modifiers of accuracy (torque, reuse/sterilization, geometry/height, scan strategy, and environmental conditions) into clinically oriented recommendations. Finally, it delineates clear research priorities, including in vivo trials, standardized reporting, and links to long-term prosthetic outcomes.

### 4.7. Limitations of This Review

It is acknowledged that, as a scoping review, the methodology did not include a formal risk-of-bias assessment or a meta-analysis; thus, while the evidence is mapped, there is no quantitative synthesis of the data. Many of the included studies are in vitro, and the reproducibility of the results for in vivo patient conditions is uncertain. The heterogeneity in study designs also limits cross-study comparability.

## 5. Conclusions

PEEK intraoral scan bodies appear to be a promising option for digital implant impressions, particularly in short-span and partial-arch restorations due to their favorable optical properties. However, their accuracy is influenced by multiple factors, including scan body design, implant position, scanner type, and torque application.

The current evidence is predominantly from in vitro studies, with limited clinical data. Therefore, although PEEK scan bodies generally demonstrate clinically acceptable accuracy, definitive conclusions regarding their clinical superiority and indications cannot yet be established. Further well-designed clinical studies are required to confirm their performance in clinical settings.

## Figures and Tables

**Table 1:** Summary of studies comparing PEEK and titanium scan bodies in terms of accuracy-related outcomes.

Author	Design	Comparison	Outcome	Result	Conclusion
Arcuri et al., 2020 [19]	In vitro	PEEK vs. titanium scan body materials	Accuracy	Scan body material influenced complete-arch digital impression accuracy	Material choice affected accuracy in complete-arch scanning
Lee et al., 2021 [18]	In vitro	PEEK vs. titanium	Trueness	Titanium showed better trueness, whereas PEEK showed a higher within-tolerance percentage	Material effects varied according to angulation and scanning conditions
Azevedo et al., 2024 [37]	In vitro	PEEK vs. plasma-coated titanium	Trueness and precision	Plasma-coated titanium generally showed higher trueness and precision than PEEK	Accuracy depended on both material and IOS type
Costa Santos et al., 2025 [38]	In vitro	PEEK vs. titanium after repeated autoclaving	Dimensional stability	PEEK showed greater deformation than titanium, although both remained within clinically acceptable limits	Both materials remained clinically usable, but titanium was more dimensionally stable
Diker et al., 2023 [39]	In vitro	PEEK vs. titanium under different torque and sterilization conditions	Displacement	PEEK displaced more than titanium, especially at higher torque and after sterilization	Titanium showed greater stability; PEEK should be used with controlled torque and limited sterilization cycles
Hashemi et al., 2023 [41]	In vitro	PEEK vs. titanium after reuse/sterilization	Dimensional stability	PEEK showed less inter-implant distance variation, but greater diameter change	Reuse affected different dimensional parameters differently according to material
Baranowski et al., 2025 [45]	In vitro	PEEK vs. titanium prototypes	Trueness	Titanium scan bodies were more accurate than PEEK	Titanium appeared more favorable in full-arch scanning, although design also influenced performance
Althubaitiy et al., 2022 [46]	In vitro	PEEK vs. titanium	Precision	Titanium performed better in desktop scanning, whereas PEEK performed better in intraoral scanning	Accuracy depended on both material and scanner type
Morita et al., 2025 [48]	In vitro	PEEK vs. titanium	Vertical displacement under torque	Titanium behaved more similarly to the titanium abutment than PEEK under higher torque	Titanium showed greater mechanical stability under increased tightening torque
Qasim et al., 2024 [54]	In vitro	PEEK vs. titanium scan bodies after repeated sterilization	Surface and chemical stability	Repeated autoclaving minimally affected titanium but caused measurable alterations in PEEK	Reuse should be more cautious for PEEK scan bodies
Tawfik et al., 2024 [59]	In vitro	PEEK vs. titanium under dry/wet conditions and different exposure heights	Inter-implant accuracy	Titanium tended to be more precise than PEEK; wet conditions worsened accuracy and greater exposure improved it	Material effects interacted with saliva and scan body exposure height
Soltan et al., 2025 [60]	In vitro	PEEK vs. titanium at 0° and 30° angulation	Trueness and precision	PEEK showed higher trueness and precision than titanium	PEEK may perform better under specific angulation and scanner conditions

**Table 2:** Summary of the main experimental and clinical factors influencing the accuracy of PEEK intraoral scan body (ISB). “Clinically acceptable” deviations were generally defined as ≤100 µm.

Influencing Factor	Main Findings	Clinical/Technical Implications
Material type (PEEK vs. titanium) [18,19,37,38,39,41,45,46,54,59,60]	PEEK shows comparable or slightly superior optical performance but lower mechanical rigidity. Titanium often exhibits higher trueness in controlled conditions.	Material choice should consider scanning environment: PEEK for optical scanning ease; titanium for mechanical stability.
Sterilization and reuse [38,39,41,42,54]	Minor dimensional changes occur after multiple autoclave cycles (≤10–50); accuracy generally within clinically acceptable limits (<100 µm). Excessive cycles increase surface wear and base deformation.	Limit PEEK ISB reuse to ≤10 cycles; inspect for deformation before each use.
Torque and seating [39,48]	High torque (>10 Ncm) causes vertical displacement and deformation in PEEK ISBs; optimal torque range is 5–10 Ncm.	Standardized torque control is critical to maintain reproducible seating accuracy.
Implant angulation and span length [18,49,50,53,60]	Minimal impact for short spans and aligned implants; deviations increase with long spans, posterior sites, or >25° angulation.	Use caution in multi-unit or full-arch cases; favor splinting or calibrated scanning protocols.
Scanner type and scanning strategy [37,45,46,47,53,60]	Scanner model strongly affects accuracy, Primescan (Dentsply Sirona, Bensheim, Germany), TRIOS (3Shape A/S, Copenhagen, Denmark), Medit i700 (Medit Corp., Seoul, South Korea) perform best). Zig-zag or segmental scanning reduces stitching error.	Select high-performance IOS systems; optimize scanning path for long spans.
Geometry and height of scan body [45,47,59]	Simple cylindrical designs and flat surfaces enhance trueness; excessive height or complex shapes may reduce accuracy.	Prefer geometrically simple, well-indexed designs with adequate supragingival exposure.
Environmental conditions [59]	Light intensity, color temperature, and saliva presence influence optical detection, particularly in full-arch scans.	Control illumination and moisture during scanning to reduce data noise and stitching errors.
Connection type and interface stability [19,40,41,43,47,56,58]	Instability at ISB–implant interface (especially in hybrid PEEK–titanium models) contributes to positional errors.	Ensure precise fit and avoid mixed-material ISBs where possible.

## Data Availability

No new data were created or analyzed in this study.

## Supplementary Materials

The following supporting information can be downloaded at: https://www.mdpi.com/article/10.3390/dj14040222/s1, Table S1: PRISMA 2020 Checklist; Table S2: Detailed characteristics and principal findings of all included studies.

## Abbreviations

The following abbreviations are used in this manuscript:

ISB	Intraoral Scan Body

## Appendix A. Detailed Search Strategies

The detailed database-specific strategies were as follows:

- PubMed (MEDLINE):

(((((((dental implant[MeSH Terms]) OR (dental implants[MeSH Terms])) OR (dental impression technique[MeSH Terms])) OR (dental implant*)) OR (intraoral scan*)) OR (digital impression*)) OR (IOS)) OR (optical impression*)

AND

(((polyetheretherketone) OR (PEEK)) OR (polymer)) OR (high performance polymer)

AND

((((((scanbod*) OR (scan body)) OR (scan bodies)) OR (scan flag*)) OR (scan* abutment*)) OR (scan post*)) OR (implant impression post*)

AND

((cad cam[MeSH Terms]) OR (CAD/CAM)) OR (digital workflow*)

- Embase:

(‘dental implant’/exp OR implant*:ti,ab,kw OR “dental implant*”:ti,ab,kw OR ‘intraoral scanner’/exp OR “intraoral scan*”:ti,ab,kw OR “intra-oral scan*”:ti,ab,kw OR ‘dental impression’/exp OR “digital impression*”:ti,ab,kw OR “optical impression*”:ti,ab,kw OR (ios:ti,ab,kw AND (intraoral:ti,ab,kw OR dental:ti,ab,kw)))

AND

(‘polyetheretherketone’/exp OR peek:ti,ab,kw OR polyetheretherketone:ti,ab,kw OR “high performance polymer*”:ti,ab,kw OR polymer*:ti,ab,kw)

AND

(“scan bod*”:ti,ab,kw OR scanbod*:ti,ab,kw OR “scanning abutment*”:ti,ab,kw OR “scan abutment*”:ti,ab,kw OR “scanning post*”:ti,ab,kw OR “scan post*”:ti,ab,kw OR “scan flag*”:ti,ab,kw OR scanflag*:ti,ab,kw OR “implant impression post*”:ti,ab,kw OR ‘impression coping’/exp)

AND

(‘computer aided design’/exp OR ‘computer aided manufacturing’/exp OR ‘computer aided dentistry’/exp OR “CAD/CAM”:ti,ab,kw OR CAD-CAM:ti,ab,kw OR “digital workflow*”:ti,ab,kw)

NOT

(spine:ti,ab,kw OR spinal:ti,ab,kw OR orthop*:ti,ab,kw OR orthopaed*:ti,ab,kw OR intervertebral:ti,ab,kw OR cage:ti,ab,kw OR tibia*:ti,ab,kw OR femur*:ti,ab,kw OR hip:ti,ab,kw OR knee:ti,ab,kw OR shoulder:ti,ab,kw)

- Scopus:

TITLE-ABS-KEY(implant* OR “dental implant*” OR “intraoral scan*” OR “intra-oral scan*” OR “digital impression*” OR “optical impression*” OR (IOS AND (intraoral OR “intra-oral” OR dental)))

AND TITLE-ABS-KEY(peek OR polyetheretherketone OR “high performance polymer*” OR polymer*)

AND TITLE-ABS-KEY(“scan bod*” OR scanbod* OR “scanning abutment*” OR “scan abutment*” OR “scanning post*” OR “scan post*” OR “scan flag*” OR scanflag* OR “implant impression post*”)

AND TITLE-ABS-KEY(“CAD/CAM” OR CAD-CAM OR “computer aided design” OR “computer-aided design” OR “computer aided manufacturing” OR “computer-aided manufacturing” OR “digital workflow*”)

AND NOT TITLE-ABS-KEY(spine OR spinal OR orthop* OR orthopaed* OR intervertebral OR cage OR tibia* OR femur* OR hip OR knee OR shoulder)
