# Supplementary material for: PEEK Intraoral Scan Bodies—A Scoping Review
Source: Dent J (Basel). 2026 Apr 9;14(4):222. doi: 10.3390/dj14040222 (PMC13115467; doi:10.3390/dj14040222)
Supplement: Supplementary file 1 [file dentistry-14-00222-s001.zip › dentistry-4217986-supplementary S2.pdf]

	Year	Study type	Sample/Jaw/Region	Type of Edentulism	No of implants	Scan body material(s)	Scanner	Method	Key findings
[32] Stimmelmayr, et al	2012	In vitro	Mandibular polymer model, stone cast	Full	4 implants(4 implant analogs in stone model)	PEEK	Lab scanner Everest Scan Pro; KaVo, Biberach, Germany	STL superimposition with inspection software (Comet INSPECT Plus 4.5); discrepancies measured at 21 points on each scanbody; statistics with Mann–Whitney U test and ANOVA	Mean discrepancy of scanbodies: 39 µm (±58) on original implants vs. 11 µm (±17) on lab analogues (statistically significant, $p < 0.05$)<br><br>Systematic error lower for stone models (5 µm) than polymer models (13 µm)<br><br>Reproducibility of fit was better on lab analogues than on original implants
[33] Giménez-Gonzalez, et al	2015	In vitro	Maxilla model	Full	6 implants	PEEK	3M True Definition	CMM reference, superimposition analysis, statistical evaluation (ANOVA, t-tests)	PEEK scan bodies provided deviations within acceptable limits (<70 µm).<br><br>Accuracy was influenced by scanbody visibility (subgingival placement decreased accuracy), scan length, and operator experience.<br><br>Implant angulation had no effect.
[34] Papaspyridakos, et al	2015	In vitro	Mandible	Full,	5 implants	PEEK	TRIOS (3Shape, Denmark)	STL superimposition with best-fit alignment	Digital impressions were as accurate as splinted conventional impressions and more accurate than non-splinted ones.

									Implant angulation up to 15° did not affect accuracy.
[35] Amin, et al	2016	In vitro	Mandible	Full	5 implant analogs	PEEK SB	CEREC Omnicam (Sirona) and 3M True Definition	STL superimposition (best-fit)	Digital impressions (TrueDef 19 µm, Omnicam 46 µm) were more accurate than conventional impressions (168 µm). TrueDef outperformed Omnicam.
[19] Arcuri, et al	2020	In vitro	Maxillary PMMA model	Full	6 implant analogs	PEEK, Ti, PEEK–Ti base	TRIOS 3 (3Shape, Copenhagen, Denmark)	Superimposition with Geomagic Studio; statistical analysis via mixed linear model	PEEK scan bodies provided highest accuracy (DASS ≈ 55 µm; angular deviation ≈ 0.64°).<br><br>Titanium performed moderately (≈99 µm).<br><br>PEEK–titanium hybrid showed worst accuracy (≈196 µm). Implant position influenced deviations; operator had no effect.
[36] Arcuri, et al	2022	In vitro	Mandibular PMMA model	Full	4 implant analogs	PEEK	TRIOS 3 (3Shape A/S, Copenhagen, Denmark)	Alignment of STL test files to reference (Geomagic Studio 12); deviations analyzed with HyperCAD-S; statistical analysis with ANCOVA (SAS, R)	ISB wear negatively influenced accuracy, particularly for angulated implant at position 3.6 (17° distal) ($p < 0.0001$)<br><br>Main deviation sources were Y-axis (lateral) and X-axis (longitudinal) shifts<br><br>Concluded that PEEK ISB base wear reduces accuracy, especially in tilted implants

[37] Azevedo, et al	2024	In vitro	Mandibular gypsum cast	Full	6 implant analogs	PEEK (White Scanmarker, Zirkonzahn), Plasma-coated medical titanium (White Metal Scanmarker, ZIrkozahn)	TRIOS 4 (3Shape), Virtuo Vivo (Dental Wings), Medit i700 (Medit), iTero 5D (Align), Primescan (Dentsply Sirona)	STL superimposition with Geomagic Control X (best-fit alignment, ISBs cut at scan region); statistical analysis with Kruskal-Wallis/ANOVA + Bonferroni correction	Significant interaction between ISB material and IOS (P = .001)<br><br>Plasma-coated titanium<br>ISBs generally had higher trueness (33 ± 6 μm) and precision (32 ± 9 μm)<br>than PEEK<br>ISBs (trueness 47 ± 27 μm; precision 40 ± 15 μm)<br>Primescan showed highest accuracy regardless of ISB material (P = .912)<br><br>TRIOS 4 had lowest accuracy with PEEK ISBs, while Virtuo Vivo had lowest accuracy with titanium ISBs<br><br>VV performed best with PEEK ISBs, while T4, Mi700, and i5D performed best with titanium ISBs<br>Both materials produced deviations < 120 μm, within clinical acceptability

[38] Costa Santos, et al	2025	In vitro	Two 3d printed mandibular models	Full	10 implant analogs each	PEEK, Titanium	InEOS X5 bench scanner (Dentsply Sirona)	SBs subjected to 0, 1, 10, 50, 100 autoclave **cycles** (134°C, 40 min) with 10 Ncm torque screwing; STL superimposition (Geomagic Control) for surface deviations; qualitative marginal fit under optical microscope (40×) at 4 surfaces; statistical analysis with ANOVA and Bonferroni correction	PEEK SBs showed greater deformation than titanium SBs, especially at implant-level after 100 cycles (≈50 μm vs ≈20 μm)<br><br>Despite deformation, all deviations remained <50 μm (clinically acceptable)<br><br>Implant-level SBs more affected than abutment-level<br><br>Microscopic analysis: 100% of SB faces classified as "clinically adapted", even after 100 cycles<br><br>Conclusion: Both PEEK and titanium SBs remained viable after repeated autoclaving, though titanium was more dimensionally stable.
[39] Diker, et al	2023	In vitro	Epoxy resin sleeves	Not applicable	2 implants	PEEK (Zfx ISB), Titanium (MPS Zimmer ISB R1410, Medentika)	No IOS used – Digital Image Correlation (DIC**) system** with dual cameras (Vic-3D software)	Torque applied at 5 → 10 → 15 Ncm using digital torque device; displacements measured via 3D DIC before and after **25 autoclave sterilization cycles**; statistical tests: Mann–Whitney U, Wilcoxon signed-rank	PEEK SBs displaced more than Ti SBs on all axes ($p < 0.05$), especially at 15 Ncm and after sterilization<br><br>Sterilization generally increased PEEK SB displacement (e.g., x-axis 40–71 μm after autoclave vs 14–39 μm before)<br><br>Ti SBs showed minimal changes (<5 μm) regardless of torque/sterilization<br><br>Authors recommend ≤10 Ncm torque for PEEK SBs,

									avoid multiple sterilizations, and consider Ti SBs for greater stability
[40] Grande, et al	2025	In vitro	Maxillary titanium model	Full	No implants, only multi-unit	ISB-AQ (sandblasted titanium, 10 mm)<br><br>ISB-One-shot (sandblasted titanium, 6 mm, reduced geometry)<br><br>ISB-PEEK (PEEK body with titanium base, 10 mm; Elos)	TRIOS 3 POD (3shape)<br><br>Medit i700 (Medit)<br><br>iTero 5D (Itero)<br><br>Primescan (Dentsply Sirona)	Two scan strategies—Zig-zag (ZZ) and One-shot (OS); superimposition by fiducials; automatic centroid/axis computation (MATLAB); ANOVA/Tukey with α=0.05	ISB-AQ (Ti) showed highest trueness, outperforming One-shot (Ti) and PEEK/Ti-base.<br><br>TRIOS had best accuracy overall; scan strategy did not affect trueness generally (except Primescan, where OS > ZZ).<br><br>Fastest scans: One-shot ISB, Primescan, and ZZ strategy.<br><br>PEEK/Ti-base produced the largest deviations among the three ISBs.
[41] Hashemi, et al	2023	In vitro	2 acrylic resin maxillary models	Partial	2 implant analogs	Two pieces Scan bodies:<br><br>Titanium scan body (Doowom, Arum, Daejeon, Korea) and PEEK scan body (NT-trading, Scanbody 3D-Guide, Karlsruhe, Germany); both had titanium connections	TRIOS (3Shape, Copenhagen, Denmark)	Each scan body type was attached (10 Ncm torque), scanned, removed, and autoclaved 9 times at 134°C for 10 min (15 min drying). STL files analyzed in GOM software (ATOS Core 80); scans superimposed with best-fit alignment; reference cube defined 3D coordinates. Statistical tests: t-test (α = 0.05).	Inter-implant distance variation was significantly greater in titanium scan bodies (0.032 ± 0.016 mm) than PEEK (0.011 ± 0.012 mm) ($p = 0.006$).<br><br>Diameter change was greater in PEEK (0.066 ± 0.014 mm) than titanium (0.029 ± 0.020 mm) ($p < 0.001$).<br><br>No significant difference in ΔR

									(0.069 ± 0.052 mm vs 0.080 ± 0.044 mm; p = 0.759).<br><br>PEEK scan bodies performed better after 10 reuse/sterilization cycles, maintaining more stable inter-implant distances.
[42] Kato, et al	2022	In vitro	2 stone casts	Not applicable	2 implant bodies and 2 implant analogs	PEEK scan bodies (CARES Mono SB RN for TL, CARES Mono SB RC for BL, Straumann)	TRIOS 3 (3Shape, Copenhagen, Denmark)	Autoclave at 135°C for 3 min; torque 15 Ncm; 10 repeated connection/disconnection cycles; STL analysis using PolyWorks Inspector (InnovMetric, Canada); SEM for surface texture; statistical tests with t-test and Tukey ($p < 0.05$)	Autoclave treatment caused small but significant deformation in distance and angle for tissue-level and bone-level PEEK scan bodies (up to ~31 µm and 0.33°).<br><br>Repeated tightening or combined autoclave plus tightening did not cause significant changes.<br><br>SEM showed minor grooves after initial connection but no progressive surface damage.<br><br>Authors concluded that PEEK scan bodies can be reused under proper sterilization without clinically relevant deformation.

[43] Kim, et al	2020	In vitro	Implants in auto polymerized resin (Orthodontic Resin)	Not applicable	1 implant fixture	4 scan body types:<br><br>3 made of PEEK:<br><br>Straumann (PEEK) Group (RN Straumann CARES Mono Scanbody, Straumann, Basel, Switzerland),<br><br>Dentium (PEEK) Group (IOS Healing Abutment, Dentium, Seoul, Republic of Korea),<br><br>Myfit (PEEK) Group (All PEEK Scanbody, Myfit, Daegu, Republic of Korea)<br><br>1 whose base made of titanium:<br><br>Myfit (Metal) Group (All PEEK Scanbody, Myfit, Daegu, Republic of Korea)]	E1 laboratory scanner (3Shape, Copenhagen, Denmark)	Each scan body was tightened by hand (mean 15.7 ± 1.3 Ncm), at 5 Ncm, and at 10 Ncm. Five scans were recorded per torque condition, for a total of N = 45 scans. STL files were analyzed in Geomagic Control X and compared to reference CAD models. Statistical analysis: ANOVA and Tukey HSD (α = .05).	Straumann (PEEK) and Myfit Metal (titanium) scan bodies showed the lowest 3D and vertical displacements, while Dentium and Myfit (PEEK) showed higher deviations.<br><br>Vertical displacement exceeded 100 µm for PEEK scan bodies under hand tightening but remained below 100 µm at 5 and 10 Ncm.<br><br>Horizontal displacement was below 10 µm for all groups.<br><br>PEEK scan bodies were more susceptible to deformation, especially under hand tightening.<br><br>The authors recommended 5 Ncm torque for optimal accuracy.

[44] Lawand, et al	2024	In vitro	1 maxillary stone cast	Full	4 implant analogs	PEEK/TAN monolithic ISBs (CARES Mono Scanbody Ø4.6 × 9 mm) in three conditions: nonmodified (NM), subtractively modified (SM; four round grooves buccal/lingual/mesial/distal), additively modified (AM; four cemented PEEK beads)	TRIOS 3 (3Shape)	15 consecutive scans per group (NM, SM, AM) under controlled ambient conditions; reference best-fit alignment on a gingival region; measurements and statistics in Geomagic Control X with repeated-measures ANOVA/Bonferroni; 1-way ANOVA for time	Significant differences among groups.<br><br>Additively modified ISBs showed the highest 3D RMS error (overall ≈0.266 ± 0.030 mm) and worse trueness;<br><br>subtractively modified ISBs yielded the lowest mean angular deviation (global ≈0.993 ± 0.062°) and generally better linear/angular trueness than NM and AM;<br><br>scanning time did not differ significantly among groups (≈1:30–1:40).
[18] Lee, et al	2021	In vitro	3 mandibular modes	Full	6, 2 implants per model (second premolar and second molar sites)<br><br>six groups created based on distal implant angulation (parallel, 15° mesial, 15° lingual)	PEEK and titanium (Myfit, Daegu, South Korea)	CS3600 (Carestream Dental), TRIOS 3 (3Shape), and Primescan (Sirona Dental Systems)	Each group scanned 10 times with each intraoral scanner (total 180 scans); best-fit superimposition to reference scan; data analyzed with Kruskal–Wallis and Mann–Whitney U tests (α = 0.05, Bonferroni correction)	Both implant angulation and scan body material significantly affected trueness ($p < .001$).<br><br>Titanium scan bodies showed better trueness (median RMS 222.1 µm) but lower within-tolerance percentage (65.7%) than PEEK (RMS 349.9 µm; within-tolerance 72.4%).<br><br>Mesially tilted implants produced the best trueness (RMS 150.5–264 µm).<br><br>TRIOS3 exhibited the best accuracy among scanners.<br><br>The authors concluded that titanium scan bodies

									yield more accurate but less tolerant scans, and that mesial angulation enhances scan trueness.
[45] Baranowski, et al	2025	In vitro	Metal mandibular cast	Full	9 implants	Control scan body: PEEK (ELOS Accurate IO-2A-B); prototypes: titanium (polished or Al_2O_3-blasted), PEEK, and variants differing in length (shorter or longer), top design (concave), and screw-hole size (100% and 200% enlarged)	NeoScan1000 intraoral scanner (Neoss, Gothenburg, Sweden)	Seven prototype subgroups scanned 10 times each under two mucosal thicknesses. Reference model digitized with 3Shape E3 desktop scanner (trueness 10–20 µm). Deviations analyzed in GOM Inspect (Zeiss) relative to fixed reference points (middle cross and first scan body). Statistical analysis with Welch test (α = .05) and Games-Howell post hoc (Bonferroni correction, p < .007).	Material significantly affected trueness: titanium ISBs (80 ± 72 µm polished; 89 ± 86 µm blasted) were more accurate than PEEK (149 ± 131 µm).<br><br>Shorter ISBs (172 ± 143 µm) showed the highest angular deviation (0.64 ± 0.70°).<br><br>Longer ISBs (248 ± 39 s) increased scanning time but did not improve accuracy.<br><br>Larger screw-hole ISBs improved usability without compromising accuracy.<br><br>Concave top ISBs enhanced trueness in deeper implants.<br><br>Titanium blasted ISBs provided the best balance of accuracy and scanning efficiency.

									Stitching errors were the main source of depth inaccuracies in full-arch scans.
[46] Althubaitiy, et al	2022	In vitro	Mandibular stone cast	Partial, missing premolars and first molar	4 implant analogs	Titanium scan bodies (Medentika REF L1400, L1410) and PEEK scan bodies (Straumann CARES Mono Scanbody REF 025.2915, 025.4915)	Extraoral scanner (E1; 3Shape, Copenhagen, Denmark) and intraoral scanner (TRIOS 3; 3Shape, Copenhagen, Denmark)	Each scanner performed 11 scans per condition (no ISB, titanium ISB, PEEK ISB); total 66 scans. Reference scan (S1) compared to 10 test scans (S2–S11). 3D superimposition performed in Geomagic Control X (best-fit alignment using teeth as reference). Nonparametric tests (Kruskal–Wallis, Wilcoxon with Bonferroni correction, $\alpha = 0.05$).	Use of ISBs reduced overall scan precision compared to the cast without ISBs.<br><br>EOS overall precision: no ISB 15.96 µm, titanium 21.68 µm, PEEK 57.57 µm.<br><br>IOS overall precision: no ISB 56.87 µm, titanium 113.05 µm, PEEK 76.16 µm.<br><br>EOS specific precision (best to worst): RD Ti > ND Ti > RD PEEK > ND PEEK.<br><br>IOS specific precision followed the reverse order: ND PEEK > RD PEEK > ND Ti > RD Ti.<br><br>EOS generally provided higher precision than IOS.<br><br>Authors concluded that scan accuracy depends on ISB material and diameter, with titanium performing better for desktop scanning and PEEK performing better for intraoral scanning.

[47] Meneghetti, et al	2023	In vitro	3d printed mandibular model	Full	6 implants	SB1 PEEK with metal connection (S.I.N., São Paulo, Brazil); SB2 PEEK (Neodent, Curitiba, Brazil); SB3 PEEK (Neodent, Curitiba, Brazil); SB4 3D-printed grey resin (prototype); SB5 3D-printed grey resin (prototype); SB6 3D-printed grey resin (prototype, bar type); SB7 PEEK (S.I.N., São Paulo, Brazil)	Primescan (Dentsply Sirona, Bensheim, Germany)<br><br>Omnicam (Dentsply Sirona, Bensheim, Germany)<br><br>TRIOS 3 (3Shape, Copenhagen, Denmark)<br><br>TRIOS 4 (3Shape, Copenhagen, Denmark)	STL exports aligned to the reference model using 3Shape Convince software; deviations analyzed in Blender (Blender Foundation, Amsterdam, Netherlands) with custom Python script; statistical analysis via Kruskal–Wallis and Bonferroni correction ($\alpha = 0.05$)	Significant differences among intraoral scanners and scanbody designs ($p < .001$).<br><br>Primescan showed the lowest median 3D deviation (110.6 μm), followed by TRIOS 4 (122.4 μm), TRIOS 3 (130.6 μm), and Omnicam (worst).<br><br>The most accurate scan bodies were SB2 (Neodent PEEK, 72.3 μm) and SB7 (S.I.N. PEEK, 93.3 μm).<br><br>Prototype 3D-printed resin scan bodies (SB4–SB6) exhibited the highest deviations.<br><br>Linear distance deviations favored Primescan and selected PEEK scan bodies, confirming that shorter, cylindrical PEEK designs with a beveled face enhance scanning accuracy.

[48] Morita, et al	2025	In vitro	Cuboid laminated bone model	Not applicable	1 implant	PEEK scan body (CARES Mono Scanbody RC; Straumann, Basel, Switzerland) and titanium scan body (L Series Scanbody RC; Medentika, Hügelsheim, Germany); titanium abutment (RC Temporary Abutment Crown; Straumann, Switzerland)	E4 laboratory scanner (3Shape, Copenhagen, Denmark)	Components were tightened to 10 Ncm and 35 Ncm using a digital torque wrench (iSD900, JMM, Osaka, Japan). STL data superimposed in PolyWorks Inspector (v18.9.6181, InnovMetric, Quebec City, Canada) with bone block planes as reference. Vertical displacement measured at the top surface of each component; Mann–Whitney U test with Bonferroni correction ($\alpha = 0.05$).	All groups showed greater vertical displacement at 35 Ncm ($p < 0.01$).<br><br>Median displacement: PEEK scan body −16.0 μm, titanium scan body −19.0 μm, titanium abutment −19.0 μm.<br><br>No significant difference between titanium scan body and abutment, but both differed significantly from PEEK ($p < 0.01$).<br><br>Authors concluded that titanium scan bodies replicate titanium abutment subsidence more accurately than PEEK scan bodies under higher torque.
[49] Nagata, et al	2021	Prospective clinical study	30 patients	Partial	50 implants	PEEK scan body (Mono Scanbody RC/RN; Straumann, Basel, Switzerland)	TRIOS 3 intraoral scanner (3Shape, Copenhagen, Denmark)	STL files from digital and conventional impressions superimposed in Geomagic Control (3D Systems, USA) after manual alignment and best-fit registration; misfit measured by averaging three points on each scan body; statistical comparison via Tukey–Kramer method ($\alpha = 0.05$)	Mean scan body misfit (μm): A (single implant) 40.5 ± 18.9; B1 (two-unit mesial free-end) 45.4 ± 13.4; B2 (two-unit distal free-end) 56.5 ± 9.6; C1 (three-unit mesial free-end) 50.7 ± 14.9; C2 (three-unit distal free-end) 80.3 ± 12.4.<br><br>Accuracy decreased with longer spans and greater distance from adjacent teeth.<br><br>Authors concluded that IOS-based impressions are clinically acceptable for implant-supported prostheses up to three units in a bounded edentulous saddle.

[50] Pan, et al	2020	In vitro	Maxillary resin model (Nobel Biocare), duplicated in gypsum	Full	6 implants	PEEK scan bodies (Zfx Evolution Matchholder, NO-MU-4.8, Zimmer Biomet, USA)	Zfx Evolution plus+ laboratory scanner (Zimmer Biomet, USA)	Ten scans per condition: (1) control scan (CMM reference), (2) scan without removing scan bodies (C), (3) scan with scan bodies removed and reinserted in the same positions (CR), and (4) scan with scan bodies removed and randomly repositioned (RR). STL data analyzed in Geomagic Control 2014 (Geomagic, Morrisville, USA); two-way ANOVA, $\alpha = 0.05$.	Mean linear distortion: C = 16.6 μm, CR = 27.6 μm, RR = 34.2 μm.<br><br>Angular deviation showed no significant differences among groups.<br><br>Repositioning scan bodies significantly reduced distance precision, especially in anterior and cross-arch regions.<br><br>Despite this, all deviations remained within clinically acceptable limits.<br><br>The authors concluded that repeated removal and random repositioning of PEEK scan bodies can decrease scan reproducibility.
[51] Pan, et al	2025	In vitro	PEEK blocks	Not applicable	No implants, Scan bodies bonded on PEEK blocks	Fifteen PEEK scan bodies fabricated from three PEEK blocks; included nine cylindrical (Ø4.8, 5.5, 6.5 mm × 4, 8, 12 mm), five cuboidal (3×6×8 mm, 3×6×12 mm, 4×6×6 mm, 5×6×8 mm, 5×6×12 mm), and one spherical (Ø8 mm) design	D2000 laboratory scanner (3Shape, Copenhagen, Denmark)	Each scan body measured three times with CMM and ten times with the lab scanner; STL data analyzed in Geomagic Control X (Geomagic, Morrisville, USA) to calculate Euclidean distance and angular deviation. Statistical analysis performed using one-way and two-way ANOVA with Tukey and Dunnett T3 post hoc tests ($\alpha = 0.05$).	Shape and size significantly influenced scan accuracy.<br><br>Cylindrical scan bodies showed superior linear accuracy (9.5 ± 6.2 μm) compared with cuboidal (17.7 ± 8.1 μm) and spherical (12.5 ± 6.5 μm).<br><br>Cuboidal scan bodies demonstrated higher angular trueness (0.050 ± 0.009°) than cylindrical (0.065 ± 0.040°).<br><br>Within the cylindrical group, narrower designs (Ø4.8 mm)

									showed inferior accuracy, while wider (Ø5.5 mm and Ø6.5 mm) and taller (12 mm) designs achieved significantly better angular trueness (p < 0.001).<br><br>Spherical scan bodies could not transfer implant angulation and were unsuitable as standalone scan bodies.<br><br>Authors concluded that longer (>8 mm) and wider (>Ø4.8 mm) scan bodies are more accurate for transferring 3D implant position.
[52] Park, et al	2024	In vitro	Two identical epoxy resin mandibular reference casts	Partial	6 implants, 3 in each cast				
[53] Pozzi, et al	2022	In vitro randomized trial	Mandibular PMMA model	Full	4 implants	PEEK scan bodies (LaStruttura Spa, Varese, Italy)	TRIOS 3 (3Shape A/S, Copenhagen, Denmark)	60 scans (30 ISS+, 30 ISS−) taken by a single experienced operator; reference model digitized using ATOS Compact Scan 5M (GOM GmbH, Braunschweig, Germany); deviations measured via Geomagic Studio 12 and HyperCAD S; data analyzed using ANOVA and multivariate models (R 3.4)	Implant position significantly affected linear and angular deviations (p<0.0001).<br><br>Posterior implants (especially 3.6 and 4.7) showed higher deviations.<br><br>Scan body splinting (ISS+) reduced linear error at position 4.7 (−60.3 µm; p=0.0188) and angular error at 3.6 (−0.2406°; p<0.0001).

									The splinted scan body technique improved complete-arch scanning accuracy, particularly in posterior regions.
[54] Qasim, et al	2024	In vitro	Not applicable	Not applicable	Not applicable	Four types of scan bodies—PEEK bone-level (PK BL), PEEK tissue-level (PK TL), titanium bone-level (Ti BL), and titanium tissue-level (Ti TL)—all from Straumann, Basel, Switzerland	FTIR (Tensor 27, Bruker Optics, Germany), XPS (ESCA Lab250xi, Thermo Scientific, USA), and optical profilometer (DCM8, Leica Microsystems, Germany).	Each scan body underwent three autoclave cycles (134 °C, 20 min, 210–230 kPa). FTIR and XPS assessed chemical changes; optical profilometry measured roughness at 16 standardized points per scan body.	PEEK TL and Ti BL showed significant surface roughness reduction after three sterilization cycles ($p < 0.05$).<br><br>PEEK TL demonstrated the most notable Ra decrease (3.52 → 1.95 µm) and highest volume loss (56% after two cycles).<br><br>FTIR revealed chain cleavage and ether bond degradation in PEEK (loss of diphenyl ether peak at 1185 cm^{-1}), while Ti spectra showed minimal change.<br><br>XPS confirmed small increases in oxygen and decreases in carbon content after repeated autoclaving, indicating mild oxidation.<br><br>Conclusion: Repeated autoclave sterilization minimally affected titanium but caused measurable surface and chemical alterations in PEEK scan bodies, particularly tissue-

									level designs. Reuse should be limited to ≤3 sterilization cycles to avoid degradation.
[55] Ren, et al	2021	In vitro	48 customized mandibular 3D-printed resin models	Partial	48 implants	PEEK short scan body (SSB, 7 mm; Link type M; MYFIT, Daegu, South Korea) and PEEK long scan body (LSB, 15 mm; Osstem Regular TS; Osstem, Seoul, South Korea).	CS3600 (Carestream Dental, Atlanta, USA) intraoral scanner for direct data capture; E1 (3Shape, Denmark) laboratory scanner for reference and indirect data capture.	STL files of the test datasets were superimposed onto the master reference model using Geomagic Control X (3D Systems, Rock Hill, USA); deviations analyzed via Kruskal–Wallis H test ($\alpha = 0.05$).	Direct digital impressions (PEEK SSB and LSB) showed significantly higher accuracy for proximal and occlusal contacts than conventional impression methods (CPC and PUC) ($p < 0.001$).<br><br>No significant difference between SSB and LSB ($p = 0.964$).<br><br>Occlusal contact accuracy was lower than proximal contact in the IOS groups.<br><br>Length of the scan body did not affect accuracy.
[56] Revilla-León, et al	2020	In vitro	Maxillary typodont (Hard gingiva jaw model MIS2009-U-HD-M-32; Nissin)	Partial	3 implant replicas	Three systems for the AM subgroups:<br>• AM-1 Elos Medtech — intraoral scan body (titanium base, PEEK body) + Elos Accurate model analogs<br>• AM-2 Nt-Trading — 3D-Guide intraoral scan body (titanium base, PEEK body) + two-piece digital implant model analog<br>• AM-3 Dynamic Abutment (Talladium) — system with intraoral adaptor and PEEK	E3 laboratory scanner (3Shape, Copenhagen, Denmark) used to digitize the typodont for AM workflows E3 laboratory scanner (3Shape, Copenhagen, Denmark) used to digitize the typodont for AM workflows	Typodont scanned with the lab scanner using each system's scan body to generate STL files; AM casts fabricated simultaneously with identical settings; implant replica positions on all casts measured by CMM and compared to the typodont reference via best-fit in CAD (Geomagic)	AM groups showed lower overall 3D discrepancy than conventional stone casts.<br><br>Dynamic Abutment had significantly better mesiodistal and buccolingual accuracy than conventional, while conventional had better apicocoronal (z-axis) accuracy.<br><br>Differences among AM systems mainly

						scan body + two-piece digital analog			affected angular accuracy; linear differences among AM systems were limited.
[57] Sami, et al	2020	In vitro	Mandibular polymer model	Full	6 implant analogs	Hexagonal PEEK scan bodies (3DSPA-8; Ritter Implants, Germany)	Four intraoral scanners tested — True Definition (3M ESPE, USA, software v5.2.1)<br><br>TRIOS (3Shape A/S, Denmark, software v1.4.7.5)<br><br>CEREC Omnicam (Dentsply Sirona, Germany, software v4.5.0)<br><br>Emerald Scanner (Planmeca, Finland, software v4.6.0)	Each scanner performed five scans (n=20 STL files). Files were imported into Geomagic Control X (3D Systems, USA), superimposed on the reference model using the "Best Fit Scenario," and analyzed for 3D deviations with tolerance limits set at ±0.01 mm and ±0.05 mm.	None of the scanners achieved >10% of points within the ±0.01 mm tolerance (Emerald <5%).<br><br>All scanners showed similar trueness and precision; no statistically or clinically significant differences were found.<br><br>Increasing tolerance (±0.05 mm) increased the apparent accuracy but masked deviation details.<br><br>3D color maps were the most effective method for visualizing deviation patterns.

[58] Shely, et al	2021	In vitro	3D-printed mandibular resin model (V-Print, SolFlex 650 × 350 printer, VOCO GmbH, Heidelberg, Germany)	Partial	1 implant analog	MIS scan abutment — titanium, two-piece, asymmetrical geometry<br><br>Alpha-Bio (AB) scan abutment — two-piece (PEEK body + titanium base), cylindrical geometry<br><br>Zirkonzahn (ZZ) scan abutment — one-piece PEEK, cylindrical geometry	Omnicam (CEREC AC, Dentsply Sirona, Milford, DE, USA) intraoral scanner.	Each scan abutment type was scanned 30 times with each scanner. STL files were superimposed using best-fit alignment (PolyWorks Inspector, InnovMetric, Canada). Deviations between lab and intraoral scans were calculated along all axes and angles.	All scan abutments showed some rotational deviation between intraoral and lab scans.<br><br>The AB (PEEK + titanium) scan abutment had the largest rotational deviation (1.04°), whereas MIS (titanium) and ZZ (PEEK) abutments showed about half that (≈0.5°).<br><br>The ZZ one-piece PEEK abutment demonstrated the smallest absolute displacement (D = 46 μm < 50 μm).<br><br>The MIS titanium abutment showed no statistically significant displacement in X and Z axes.<br><br>Differences likely due to geometry, material, and one- vs two-piece design.

[59] Tawfik, et al	2024	In vitro	Six 3D-printed mandibular resin models (PROSHAPE MODEL 3D PRINTING 405 nm UV resin; Creality HALOT, UK)	Full	24 implants, 4 implants per cast	PEEK ISBs and titanium ISBs (same manufacturer; Ti with two flat faces, PEEK with one flat face)	Medit i700 intraoral scanner (Medit Corp., Seoul, South Korea)	Each cast scanned under four conditions per material: dry/wet and 2 mm/4 mm exposure (total 8 scans per cast → 48 scans overall). Interimplant distances measured in Medit Design 3.1.0; statistics with Student's t-test (α = 0.05)	Longer ISBs (greater exposure) produced smaller mean differences (higher accuracy).<br><br>Wet condition increased discrepancies vs dry.<br><br>Material effect was partly position-dependent: several distances showed significantly better results for titanium versus PEEK, while some (e.g., CD, AD) were not significant.<br><br>Overall, authors concluded saliva worsens accuracy, longer ISBs improve it, and titanium ISBs tended to be more precise than PEEK under the tested conditions.
[60] Soltan et al	2025	In vitro	3D-printed maxillary model covered by 2 mm silicone artificial gingiva to simulate clinical conditions.	Full	4 implants	Titanium ISBs (80610226, 3Shape, Copenhagen, Denmark) and PEEK ISBs (IO 2B-B SA, Elos Medtech, Gothenburg, Sweden)	Lab scanner Everest Scan Pro; KaVo, Biberach, Germany	4 × 2 factorial design — 4 IOS × 4 ISB configurations (Ti 0°, Ti 30°, PEEK 0°, PEEK 30°); n = 10 scans per group → 160 datasets. Scans aligned in Geomagic Control X using ICP algorithm. Non-parametric tests (Friedman, Kruskal-Wallis with Bonferroni correction, α = 0.05).	ISB configuration, angulation, and IOS type significantly affected accuracy ($P < 0.001$).<br><br>PEEK ISBs achieved higher trueness (mean RMS 0.019–0.060 mm) and precision (0.019–0.045 mm) than titanium (0.037–0.092 mm and 0.024–0.064 mm).<br><br>PEEK 30° gave the best trueness; PEEK 0° the best precision.<br><br>Angulation improved trueness for PEEK but not titanium.<br><br>Primescan and Trios 3 outperformed

									Aoralscan 3 and Fussen S6000.
